# Donor-derived viral infections: the usual suspects and the new kids on the block

**DOI:** 10.3389/ti.2026.17050

**Published:** 2026-09-10

**Authors:** Maddalena Peghin, Paolo Antonio Grossi

**Affiliations:** University of Insubria, Varese, Italy

**Keywords:** donor derived infection/transplant infection, HHV-8, HIV, respiratory viral infections, viral hepatitis

## Abstract

The expansion of the transplant donor pool, driven by the need to reduce the disparity between supply and demand, carries the risk of use of marginal donors with viral infections, including classic viruses and emerging threats. This narrative review analyzes these challenges, including new opportunities in managing well known viruses such as Hepatitis B, Hepatitis C and Human Immunodeficiency Virus in both donors and recipients, thanks to new effective antivirals. Furthermore, we highlight the importance of monitoring emerging viral pathogens, such as Hepatitis E and Human Herpesvirus 8 (HHV-8) implementing both targeted pre-transplant donor screening and timely post-transplant diagnosis in the recipient, enabling earlier therapeutic intervention and potentially reducing recipient morbidity and mortality. Moreover, respiratory viral infections have gained significant attention as potential causes of donor derived infection (DDI) transmitted to the recipient, particularly within specific widespread and seasonal contexts, and require further attention and investigation. Continuous updates to surveillance and screening protocols are essential to optimize outcomes of recipients, ensuring transplant safety.

## Introduction

The number of individuals awaiting transplant continues to overcome donor availability. Several efforts are ongoing to expand the donor pool to include donors at increased infectious risk [1, 2].

A paradigm shift has occurred in the utilization of organs from HBV- and HCV-positive donors, with increasing acceptance of their use in both infected and uninfected recipients. The opioid epidemic has climbed sharply in specific settings (primarily North America), ascending rates of drug abusers with overdose-related deaths has led to the use of available organs from young donors with limited comorbidities, but with higher risk of transmission of HCV, HBV and Human Immunodeficiency Virus (HIV) [3–5]. Of interest that a significant increase in mortality waitlist has been described for individuals who decline these marginal organs compared with those who accept the risk reducing wait time [6, 7].

The potential for donor-derived transmission of previously underestimated viruses (Hepatitis E, Human herpesvirus 8) highlights the importance of surveillance and screening protocols in specific geographical settings [8, 9]. Respiratory viral infections have received increased attention in recent years, including as a potential cause of donor derived infection (DDI), especially in specific seasonal settings [10]. The complex and intricate relationship between climate change, human migration, and infectious disease transmission presents a global challenge that can impact the risk of DDI [11, 12].

The aim of this narrative review is to offer an update on old and new challenges in managing donor derived viral infections. DDI caused by Arboviruses will be discussed in a separate review focused on risk for infectious tropical disease transmission.

## Hepatitis C

In the past, organs from HCV positive donors were commonly discarded or restricted to recipients with chronic HCV due to poor outcomes with interferon-based therapy and high risk of transmission to uninfected recipients. The advent of effective direct-acting antivirals (DAA) has led to HCV eradication and a shift toward the use of HCV NAT positive organs. Moreover, interest in using HCV-positive donors has grown in association with the opioid epidemic in specific geographical settings, reflecting a significant expansion of the donor pool [13].

The risk of transmission of the virus varies significantly. The transmission rates among all HCV seropositive liver allografts (HCV Ab positive, HCV RNA negative) may be relatively low ranging from 9% to 16% [14]. HCV viremic donors (HCV Ab positive, HCV RNA positive) transmit HCV almost universally [15].

Optimal follow up, DAA regimen and timing of therapy initiation, has not been established and factors such as genotype, renal function, drug-drug interactions and drug access should be considered [16].

Initiation of empiric therapy with pangenotypic regimens (Glecaprevir/Pibrentasvir or Sofosbuvir/Velpatasvir) is strongly favored, because the donor genotype is usually unknown at the time of organ allocation and these regimens feature a high barrier to resistance, which successfully overcomes potential baseline mutations, that are more common in Genotype 1a and Genotype 3. Once post-transplant testing confirms the specific genotype, clinicians can consider to safely switch the patient to targeted, genotype-specific options on the basis of drug interaction profiles and local cost-effectiveness [17]. In seronegative patients who receive seropositive viremic allografts, detectable HCV RNA levels as early as 3 days post-transplant have been observed [15]. Peri transplantation (before or soon after transplant within the first week) DAA therapies, that fully prevent the development of viremia, are likely to further mitigate any HCV-associated risks for adverse outcomes, but delayed treatment (after detectable HCV RNA or at 1 month after transplant) and shortened duration approach is common and might be feasible [18, 19]. HCV surveillance and treatment protocol for the recipients of HCV Ab positive, HCV RNA negative/positive donors based on expert opinion are summarized in Table 1.

**TABLE 1:** HCV surveillance and treatment protocol for the recipient of HCV Ab positive, HCV RNA negative/positive donors for liver and non-liver organs.

Donor	Recipient
​	• HCV Ab (+), HCV RNA (−) • HCV Ab (−), HCV RNA (−)
HCV Ab (+), HCV RNA (+)	• Baseline recipient HCV RNA before transplant • check HCV RNA early after transplant (3–7 days after transplant) • Repeat HCV RNA frequently (weekly or per center protocol) up to 4–6 weeks after transplant • Start DAA therapy with a prophylactic or preemptive strategy as soon as the recipient is clinically stable and able to take oral medications • Continue treatment for the recommended course and check HCV RNA every 4 weeks until confirm that sustained virologic response at 12 weeks is achieved
HCV Ab (+), HCV RNA (−)	• Baseline recipient HCV RNA before transplant • check HCV RNA after transplantation at 1- 3 and 6 months • If HCV RNA positive: Start DAA therapy

Abbreviations: HCV, ab, HCV: Hepatitis C Virus antibody; DAA, Direct-Acting Antiviral; RNA, ribonucleic acid.

However, despite a clear decrease in discard rates, HCV positive allografts remain an underutilized resource, especially for liver transplantation [20]. Main barriers include insurance consent for DAA therapy, that is a primarily a challenge in specific countries like United States, or logistical issues through national centralized or local procedures [21]. Current studies have demonstrated similar short-term and long-term outcomes in patients transplanted with HCV viremic donors with sustained virologic response after DAA therapy, compared to non-HCV donors, even for patients undergoing re-transplantation and multivisceral transplantation [22–26]. Recent studies on HCV discordant liver transplants demonstrated the excellent survival of these organs, with low rates of relapse and similar graft survival regardless of HCV viremia or allograft fibrosis [27, 28]. Although late recurrence of HCV is rare with modern DAA, it remains a threat to necessitating surveillance for potential relapse.

Altogether current literature shows a significant increase of HCV D+/R− transplantation, underscore the safety and cost-effectiveness of this practice and support the expansion of this practice further [29, 30] However, it is essential to continue to provide full informed consent and ensure adequate access to DAA therapy in the peri-transplant period.

## Hepatitis B and hepatitis D

HBV positive donors are another potential opportunity to expand the donor pool. However, organ discard is frequent due to concerns for recipient HBV infection post-transplant and outcomes.

The risk of transmission must be stratified based on donor and recipients HBV serologic status as well as the organ transplanted, with liver being at higher risk [31, 32]. Transplantation into a recipient without serological evidence of prior HBV infection or vaccination carries the highest risk of transmission. Therefore, administration of HBV vaccination before transplantation is strongly recommended, but vaccination rates fell short of recommendations [33]. Moreover, effective nucleos(t)ide analog (NA) therapies are being used as posttransplant prophylaxis to decrease the rate of transmission depending on the donor and recipient serological profile (Table 2). Currenltly tenofovir disoproxil fumarate, tenofovir alafenamide, and entecavir are currently preferred over lamivudine due to their higher genetic barrier to resistance and to their superior antiviral potency [34].

**TABLE 2:** HBV surveillance and treatment protocol for the recipient of HBV positive donors according to recipient serological status.

Donor	Recipient	Recipient	Recipient
HBcAb positive HBsAg negative	• HBsAg (−); HBsAb (−); HbcAb (+) • HBsAg (−); HBsAb (+) ° *; HBcAb (−) • HBsAg (−); HBsAb (−); HBcAb (−)-	• HBsAg (−); HBsAb (+) °; HBcAb (+)	• HBsAg (+)
	Prophylaxis and monitoring	Prophylaxis and monitoring	Prophylaxis and monitoring
	Liver • NA lifelong prophylaxis • Every 3 months during the first year: liver function, HBsAg, HBV DNA • Every 3–6 months indefinitely: liver function, HBsAg, HBV DNA, liver function • Every 6–12 months: liver ultrasound + AFP	Liver • NA lifelong prophylaxis or according to center policy • Every 3 months during the first year: liver function, HBsAg, HBV DNA • Every 3–6 months indefinitely: liver function, HBsAg, HBV DNA, liver function • Every 6–12 months: liver ultrasound + AFP	Liver • NA lifelong ± HB Ig (short § or long‐term ^) • Every 3 months during the first year: liver function, HBsAg, HBV DNA • Every 3–6 months indefinitely: liver function, HBsAg, HBV DNA, liver function • Every 6–12 months: liver ultrasound + AFP
	Non liver organs • No prophylaxis • Every 3 months during the first 6 months: liver function, HbsAg, HBV DNA • Every 6 months later on after 6 months post-transplant: liver function, HBsAg, HBV DNA, liver function	Non liver organs • No prophylaxis or according to center policy • Every 3 months during the first 6 months: liver function, HBsAg, HBV DNA • Every 6 months later on after 6 months post-transplant: liver function, HBsAg, HBV DNA, liver function	Non liver organs • NA lifelong +/- HBIg intraoperatively • Every 3 months during the first 6 months: liver function, HBsAg, HBV DNA • Every 6 months later on after 6 months post-transplant: liver function, HBsAg, HBV DNA, liver function • Every 12 months: screening for HCC with abdominal ultrasound and AFP
HBsAg positive HDV-RNA negative	HBsAg (−); HBsAb (+) °; HBcAb (+) HBsAg (−); HBsAb(+) ° *; HBcAb (−)	HBsAg (−); HBsAb (−); HBcAb (−) HBsAg (−); HBsAb (−); HBcAb (+)	HBsAg (+) HDV negative
	Prophylaxis and monitoring	Prophylaxis and monitoring	Prophylaxis and monitoring
	Liver • NA lifelong + HB Ig (short = or long‐term ^) • Every 3 months during the first year: liver function, HBsAg, HBV DNA • Every 3–6 months indefinitely: liver function, HBsAg, HBV DNA, liver function • Every 6–12 months: liver ultrasound + AFP	Liver • NA lifelong + HB Ig (short = or long‐term ^) • Every 3 months during the first year: liver function, HBsAg, HBV DNA • Every 3–6 months indefinitely: liver function, HBsAg, HBV DNA, liver function • For liver: Every 6–12 months: liver ultrasound + AFP	Liver • NA lifelong DO + HB Ig (short = or long‐term ^) • Every 3 months during the first year: liver function, HBsAg, HBV DNA • Every 3–6 months indefinitely: liver function, HBsAg, HBV DNA, liver function • Every 6–12 months: liver ultrasound + AFP
	Non liver organs • NA 6–12 months + HB Ig (short) • Every 3 months during the first year: liver function, HBsAg, HBV DNA • Every 3–6 months indefinitely: liver function, HBsAg, HBV DNA, liver function	Non liver organs • NA 6–12 months + HB Ig (short) • Every 3 months during the first year: liver function, HBsAg, HBV DNA • Every 3–6 months indefinitely: liver function, HBsAg, HBV DNA, liver function	Non liver organs • NA lifelong DO + HB Ig (short = or long‐term ^) • Every 3 months during the first year: liver function, HBsAg, HBV DNA • Every 3–6 months indefinitely: liver function, HBsAg, HBV DNA, liver function

Abbreviations: AFP, alpha fetoprotein; anti-HBc, Hepatitis B anti-core antibody; anti-HBs, Hepatitis B surface antibody; HBsAg, Hepatitis B surface antigen; HBV, hepatitis B virus; HCC, hepatocellular carcinoma; NA, nucleos(t)ide analog.

Symbols: ° (title >10 mlU/mL); *vaccinated; § short: 1–7 days; ^ long term: one year-lifelong for HBV-HIV, or HBV-HDV, coinfected patients. To maintain an HBsAb titer of 50–500 IU/mL, depending on the recipient’s risk level.

Liver transplantation from HBV core antibody (antiHBc Ab) donor carries a high risk (on average around 50%) of transmitting hepatitis B to the recipient. A serological profile indicative of previous contact with HBV is preferable in the recipient. However, liver transplantation in HBsAg/HBsAb negative patients is possible, but prophylactic treatment and constant post-transplant monitoring is recommended. Similar short- and long-term graft and patient survival rates have been observed when compared with HBcAb negative grafts [5, 35].

Non hepatic transplantation of HBV core positive organs does not pose any additional risk to patients with a serological profile indicative of previous contact with HBV. Non liver transplantation from antiHBc Ab positive donors in HBsAg/HBsAb negative patients poses a particularly low risk of transmitting HBV to the recipient, but this risk is not absent and deserve monitoring and/or prophylaxis according to the center policy [35, 36].

Use of organs from donors with HBV infection, defined as detection of hepatitis B surface antigen (HBsAg) or detectable HBV DNA is a potential opportunity to increase organ availability, including in naïve non vaccinated recipients [37, 38]. Successful liver transplants have been performed using HBV-infected donors with similar outcomes as HBsAg negative liver grafts without excess mortality, graft loss, or post-transplant HBV-related complications. There are limited studies reporting on the safety and outcomes using HBV-positive donors for non-hepatic organ transplants, but strongly supporting this practice [39]. Of note that fulminant hepatitis in recipients of hepatic and non-hepatic grafts has been described [38, 39].

Quantifying HBV viral loads has proven to support optimization of recipient risk stratification and management in the prevention of DDI [39]. There is a lack of standardized antiviral prophylaxis and long term follow up, that should be personalized based on expert opinion (Table 2). Post-transplant monitoring of liver function, HBsAg/HBcAb seroconversion and HBV quantitative detection is recommended. Pre-transplant HBV vaccination is strongly recommended. Transplant recipients of HBsAg+ organs should receive human HBV immunoglobulin (Ig) against HBsAg, starting during the intraoperative phase, in association with a high barrier NA regardless of the immune status. The initial objective is to maintain an anti-HBs titer >50–500 IU/L, depending on the recipient’s specific risk stratification for recurrence The duration of HB Ig therapy varies according to the transplanted organ and the presence of other coinfections, since recipients of non-liver organs from HBsAg-positive donors generally require short (1–7 days) HB Ig maintenance, whereas long-term (at least 1 year) or lifelong HB Ig combination therapy remains the standard of care for liver transplant recipients, particularly those with HIV/HBV or HBV/HDV coinfection (Table 2) [40, 41].

Hepatitis D virus (HDV)/HBV coinfection merits further comments. HDV is a small defective RNA virus that requires the surface antigen of the hepatitis B virus for its replication, assemblage, and transmission. HDV donor screening is recommended in HBsAg positive donors, whereas the presence of HBV-DNA alone in the absence of HBsAg does not require screening for HDV virus. HDV infection is documented by a positive HDV antigen or positive anti-HDV IgM (a negative HDV IgM result does not exclude chronic HDV infection) or anti-HDV IgG with a titer >1:100. If anti-HDV is positive, follow up with HDV RNA testing is recommended to confirm active infection. Organ allocation must be done on the basis of donor and recipient screening due to the risk of HDV superinfection or recurrence (Table 3) [42]. HDV/HBV-coinfected liver transplant recipients do not represent a risky population compared with HBV infected patients in general, but further data on use of HDV/HBV positive organs in HDV/HBV positive recipients are needed [43]. Currently there is no approved treatment for HDV after transplant due to poor Interferon efficacy and limited role of bulevirtide in the context of liver transplantation. As thing stand now, the most effective method for preventing HDV infection of transplanted liver in these patients is dependent on preventing HBV recurrence, with an indefinite combination of NAs with HB Ig. [44, 45]. HBV vaccination remains the cornerstone of prevention of HBV and HDV [46].

**TABLE 3:** Organ allocation in HDV/HBV coinfected donors and recipients.

Donor	Recipient	Recipient
​	• HBsAg positive • HDV RNA positive	• HBsAg positive • HDV RNA negative
HBsAg positive HDV RNA positive	transplant allowed	transplant of any organ not recommended: Risk of fatal HDV superinfection
HBsAg positive HDV RNA negative	liver transplant not recommended: Risk of HDV hepatitis recurrence	transplant of any organ allowed

In conclusion the use of antiHBc and HBsAg+ grafts was is and feasible and might play an important role in expanding donor organs especially in area of high prevalence of HBV infection. However, more data are needed to study the long-term risk of DDI HBV infection and define the optimal management strategies.

## Hepatitis E

Hepatitis E virus (HEV) is an RNA virus and a major cause of acute hepatitis worldwide. HEV has 8 genotypes, but most infections are due to genotypes HEV-1 and HEV-2, that are associated with fecal-oral route via contaminated drinking water in low-income countries and to HEV-3 and HEV-4, that are related with foodborne zoonotic infections in developed regions. HEV-3 is the dominant strain in Europe and the Americas, while HEV-4 predominates in Asia (China).

In immunocompetent hosts, HEV typically causes a self-limited acute hepatitis, whereas in SOT recipients, HEV infection can cause chronic hepatitis (defined by replication beyond 3 months after infection), that can occur in around two-thirds of infected SOT. Moreover, HEV in SOT can lead to cirrhosis, graft dysfunction or rejection [47]. Persistent hepatitis E occurs primarily involving HEV-3 and 4 [48].

Transmission of HEV through solid organs is very efficient (especially for higher plasma viral load > 10^3^ IU/mL), particularly through liver grafts and highlights the importance of surveillance and screening protocols [5]. The decision to implement universal organ donor and recipient HEV screening depends on HEV incidence, since in regions with low baseline HEV prevalence routine universal screening yields minimal impact on overall transplant safety [9, 49, 50].

The screening method of choice includes both serology (HEV IgM, IgG) and PCR testing. HEV-RNA is more sensitive than serologic testing [51]. Undetectable HEV-RNA in donor serum would indicate low risk for HEV transmission, although liver grafts may harbor infectious HEV RNA even in the absence of systemic markers of infection [51]. Donor testing result for HEV infection might be done pre- or post-transplant. Detection of HEV RNA in donor plasma is not a contraindication for organ donation, but triggers improved recipient management. Managing strategies for donor derived HEV infection is based on early detection of infection (HEV-RNA weekly for the first month) and reduction of immunosuppression (especially mammalian target of rapamycin (mTOR) inhibitors and tacrolimus). In addition, for patients without spontaneous viral clearance after 1 month, a 3-month course of ribavirin is recommended, achieving sustained virologic response in approximately 80% of cases (Table 4) [52].

**TABLE 4:** Management of recipient based on donor and recipient serological and virological Markers of Hepatitis E.

Donor	Management of the recipient
HEV IgM or IgG positive HEV RNA negative	• Accept organs • Post-transplant surveillance of the recipient: HEV-RNA every 2–3 months for the first year after transplant
HEV RNA positive	• Consider accepting organs • Post-transplant surveillance of the recipient: HEV-RNA weekly for the first month • If HEV-RNA positive in the recipient (after 1 month) o Liver test at month 1; HEV-RNA in serum and stool at month 2 and 3 o Pre-emptive ribavirin soon after transplant (starting 1–3 months after transplant depending on individual risk factors * o Change of immunosuppression, if possible • If HEV-RNA negative in the recipient (after 1 month) oHEV serology after 3 months
Recipient	
HEV IgM or IgG positive and HEV-RNA negative	• Proceed with listing and transplantation • Post-transplant surveillance of the recipient: HEV-RNA every 2–3 months for the first year after transplant
HEV RNA positive	With normal liver function: • Proceed with active transplant listing • Monitor HEV RNA • Plan for post-transplant ribavirin therapy if needed Without normal liver function • Delay transplantation if possible • Prioritize early ribavirin administration prior to the transplant • Change of immunosuppression, if possible

*Risk factors to consider: liver transplant, marginal liver graft, underling liver disease, polyclonal antibodies induction.

Vaccination against HEV represents a promising prophylactic tool, though availability remains geographically restricted (licensed in China and Pakistan) [53].

## HIV

Overall, there is increasing and solid literature supporting transplantation in people with HIV [54]. However, opportunities exist to improve knowledge and overcome misconceptions about SOT for people with HIV. At the European level, the use of organs from HIV D+ is legally allowed only in 6 out of 35 (17%) countries and there is a high willingness to participate in an HIV D+/R+ among experts in the field [55]. Moreover, awareness of the ability of patients with HIV to both receive and donate organs is limited [56]. Since the 2015, HOPE Act prospective studies have demonstrated that kidney and liver transplantation from HIV D+/R+ recipients are safe and effective, with similar graft and patient survival to HIV D−/R+ transplantation. Moreover donor-derived HIV superinfection in recipients of HIV D+/R+ transplants is rare, and the clinical ramifications appear negligible [57].

A recent retrospective small experience after heart transplantation in HIV-positive recipients using HIV-positive or HIV-negative donors (4 HIV D +/R+, 6 HIV D -/R+) found no significant differences in short-term survival, rejection, infection, heart graft function and HIV suppression between both groups at 6 months. Moreover, the lack of difference in donor-derived cell free DNA provided reassurance that use of hearts from HIV D+ did not trigger increased myocyte injury [58]. This early experience provides evidence and the inclusion of cardiothoracic patients in trials might be a necessary evolutionary step [59, 60].

Prior to 2017, transplants from HIV-positive donors to HIV-negative recipients occurred only inadvertently, because the donor’s positive HIV status was discovered after the organs had been transplanted. In 2017, the first intentional liver transplantation from a living HIV-positive mother to an HIV-negative child was performed. Multidisciplinary evaluation, research trial approval and specific consent to the procedure were obtained [61]. The recipient started antiviral therapy before the transplantation to minimize the risk of HIV transmission, continued it after transplantation. No plasma or cell-associated HIV-1 DNA or RNA was detected at any stage in the recipient, although the child’s HIV status was uncertain [61].

Thanks to modern highly effective antiviral therapy, the use of organs from deceased donors with HIV for HIV-negative recipients might become a medical consideration in the future, allowing to expand the donor pool. However, this approach requires strict precautions against transmission and deeply informed, voluntary patient consent to manage the remaining ethical and clinical complexities [59, 60].

## Human herpesvirus 8

Human herpesvirus 8 (HHV-8) is an emerging topic in DDI. HHV-8 is a double-stranded DNA virus, whose life cycle is similar to that of other herpesviruses. Primary infection is usually asymptomatic in immunocompetent hosts. However, HHV-8 primary infection or reactivation in immunocompromised hosts can lead to a spectrum of diseases, ranging from malignancies (Kaposi Sarcoma- KS-, Primary Effusion Lymphoma, Diffuse Large B-cell Lymphoma and Multicentric Castleman Disease) to severe inflammatory syndromes (Kaposi Sarcoma Herpesvirus Inflammatory Cytokine Syndrome, KICS).

HHV-8 prevalence varies with geography and ethnicity. Variable ranges have been observed in North Europe (5%), United States (3%–7%), the Mediterranean (20%–30%) and Sub-Saharan Africa (> 50%). Moreover, seroprevalence is influenced by behavioral risk, being significantly increased in people living with HIV, especially in men who have sex with men (MSM) [62]. In particular a recent study performed in Unites States found a higher prevalence of HHV-8 among kidney donors with HIV compared with non-HIV (25.2% and 7.5%, respectively), particularly among MSM [63].

In the context of SOT, HHV-8 might cause a DDI, whose timing, clinical picture and severity depends on several factors. D+/R-serological status might favor primary infection that is usually associated with inflammatory syndromes and aggressive visceral forms of KS. Moreover, the degree of immunosuppression and lung and liver transplants tend to be associated with more severe disease forms and poorer outcome.

Implementation of universal HHV-8 screening for donors and recipients should be primarily driven on one hand by local HHV-8 incidence and resource availability and on the other hand by the disproportionate severity of donor-derived post-transplant infections. A targeted screening strategy based on donor risk factors could balance safety while avoiding the economic burden of universal screening. In non-endemic areas, HHV-8 testing could be prioritized for donors presenting specific epidemiological or behavioral risk profiles, such as a history of recent incarceration, injection drug use, high-risk sexual behavior, HIV coinfection or origin from highly endemic regions [62]. In addition, there is a lack of standardized screening since Enzyme-Linked Immunosorbent Assay (ELISA) has a poor performance and may miss antibodies that target specific latent-phase proteins, whereas Indirect Immunofluorescence Assay (IFA) is the preferred approach but is operator dependent [62].

Greater awareness of risk through pre-transplant screening could support accurate and timely diagnosis, enabling earlier therapeutic intervention and potentially reducing morbidity and mortality associated with HHV-8 DDI. Strict clinical and molecular monitoring is strongly recommended for high-risk patients (HHV-8 mismatch D+/R- or R+). In addition, in case of persistent HHV-8 DNAemia and symptomatic infections, switch to an immunosuppressive regimen by replacing calcineurin inhibitors with mammalian target of rapamycin (mTOR) inhibitors and antiviral treatment (foscarnet or cidofovir, that are preferred over ganciclovir) are recommended, although use of antivirals is debatable in this setting. In the case of KICS, rituximab might be additionally provided (Table 5) [8].

**TABLE 5:** Recommended monitoring and intervention for HHV-8 high risk recipient (HHV8 D+/R–and R +).

Donor and recipient	Monitoring	Treatment
HHV8 D+/R– HHV8 D+/- and R+	HHV8 DNA test • Every 15 days for the first 3 months • Monthly between the 4th and 12th month • From the 12th month onwards, in anti-HHV-8 positive recipients repeat HHV-8 DNA testing during routine check-ups and during treatment for acute rejection/intensification of immunosuppression • Skin self-examination for detection of KS-like lesions	Primary infection or reactivation with persistent positive HHV-8 DNA and symptomatic infection • Start an antiviral (foscarnet or cidofovir preferred over ganciclovir) • Replacement of CNIs with mTOR inhibitors, when feasible • If confirmed KICS, additional rituximab • If confirmed KS: use Liposomal Doxorubicin • If confirmed MCD, PEL: follow specific treatment guidelines

Abbreviations: CNIs, calcineurin inhibitors; DNA, deoxyribo nucleic acid; KS, kaposi sarcoma; KICS, kaposi sarcoma inflammatory cytokine syndrome; mTOR, mammalian target of rapamycin; MCD, multicentric castleman disease; PEL, primary effusion lymphoma.

## Respiratory viruses

Respiratory viruses have been largely unknown and underestimated as a cause of DDI. The SARS-CoV-2 pandemic and the increased use of molecular testing have raised awareness of the role of respiratory viruses in respiratory tract infections, including in the context of DDI [64–66].

In general, transplantation of non-lung organs from donors with active respiratory viral infection is considered possible and well tolerated, without transmission, except in case of organ specific contra-indications (for example myocarditis, hepatitis). Since major transplant societies offer limited guidance, the utilization of these organs is determined by individual centers, leading to significant variations in practice at both national and international levels. In contrast, lung and bowel recipients remain uniquely vulnerable due to the high likelihood of direct viral transmission in the respiratory or gastrointestinal tracts, depending on the type of virus. In general lungs should not be used from donors with proven respiratory viral infection as the primary cause of death or causing clear lower respiratory tract involvement. However, since molecular testing detects viral RNA/DNA even in the absence of active, infectious virions, this approach is associated with the risk of wasting organs that could otherwise be safely transplanted [67].

Most available literature is focused on SARS-CoV-2 [10]. As thing stand now, current strategies for donor evaluation still include universal microbiologic screening with standard SARS-CoV-2 PCR of lower respiratory tract 24–72 h before organ procurement in most countries worldwide [68, 69]. Because of the absence of the risk of transmitting SARS-CoV-2 through non-lung solid organs, some experts propose that universal, routine testing of asymptomatic, non-lung deceased donors should be re-evaluated [68]. Of interest that a recent Italian study found that molecular testing for SARS-CoV-2 RNA on 64 graft biopsies (liver and kidney) and 68 perfusion fluid samples at procurement were negative in 100% of cases, including in donors with lower respiratory tract samples with a high viral load (Ct value <30) [70]. Lung transplantation from SARS-CoV-2 is generally contraindicated, but careful consideration to proceeding might be done based on risks and benefits, generally provided that lower respiratory tract testing is negative and antivirals are administered to the recipient [67, 71].

The use of organs from donors with positivity for Rhinovirus/Enterovirus, seasonal Coronavirus, Influenza, Paramyxovirus (Parainfluenza Virus, Human Metapneumovirus-MPV-, Respiratory Syncytial Virus-RSV-), Adenovirus and Boacavirus is a challenging topic, due to limited available data [72–74]. Screening for respiratory viral pathogens other than SARS-CoV-2 at donor evaluation is not recommended routinely and is usually conducted only in symptomatic donors. Moreover, deepening on guidelines, screening might be limited only to Influenza virus during epidemic periods or might include a complete multiplex PCR panel [75]. Therefore, the true incidence and burden of these diseases remain poorly understood. Certain viruses, namely Influenza and those from the Paramyxovirus are more likely to be associated with lower respiratory tract infections, higher hospitalization rates compared with Rhinovirus/Enterovirus and seasonal Coronavirus in lung transplant recipients [76]. Particular attention should be paid to Adenovirus-positive donors, given the virus propensity to cause systemic dissemination or end-organ damage in immunocompromised recipients depending on the virus serotype and the organ involved and to influenza A/H5N1 for the higher risk of viremia [73, 77]. However, there is limited reported evidence to support systematic viral testing of donor respiratory specimens, with a concrete risk of difficult to interpret result and discard of low-risk donors.

Most available literature on risk of DDI is based on influenza virus. There is currently no evidence demonstrating transmission of the virus through transplantation of non-lung organs, nor evidence of increased morbidity among recipients of these organs [69]. There are 2 reports of possible transmission of seasonal influenza A and B viruses by transplantation of lungs and kidneys from infected donors, but detailed molecular proof was lacking [78, 79]. Some experts recommend that recipients of organs from influenza positive donors should receive antiviral treatment for 5–10 days following transplant, unless the donor has already received a full course of treatment to reduce severity and infectivity [80]. Of note that a recent case report described a case of fatal acute respiratory distress syndrome caused by donor-derived human MPV after lung transplantation from an asymptomatic donor, confirmed after next-generation sequencing of MPV genomes [81]. Further studies are essential to precisely quantify the transmission risks of respiratory viruses during lung transplants to refine diagnostic and screening protocols.

Maintaining up-to-date vaccinations for circulating respiratory viruses in transplant candidates is crucial to mitigating risk. In general, recipients should be protected with vaccination against SARS-COV-2, influenza and RSV [82].

## Conclusion

Balancing the transmission risk of viral DDIs against the high risk of waitlist mortality is crucial, because of the growing ethical imperative to ensure equitable transplant access. By comprehensively understanding donors at risk for viral infections and their potential clinical consequences, transplant teams can perform targeted mitigation strategies, through rigorous pretransplant and posttransplant microbiological testing and target therapies.

However, the current clinical landscape is significantly limited by a lack of standardized consensus screening and management protocols for old and emerging risk viruses. Future research priorities in DDI viral infections should focus on improving the current use of post-transplant quantitative molecular testing and on exploring the role of post-exposure prophylaxis against preemptive treatment strategies in recipients. Moreover, clinical trials are urgently required through evaluating the role new advanced diagnostics, including rapid metagenomic next-generation sequencing, and new novel therapeutics, as well as virus-specific T-cell immunotherapies. Finally, coordinated financial and infrastructural investments are needed to strengthen epidemiological vigilance registries and communication networks to ensure rapid trace-backs investigations.

## References

[1] Graziano E Peghin M Balsamo ML Givone F De Martino M Montemurro A Donors infectious risk stratification: activity of the Italian national transplant center. Transpl Infect Dis (2026) 28(1):e70131. 10.1111/tid.70131 41201889

[2] Grossi PA Wolfe C Peghin M . Non-standard risk donors and risk of donor-derived infections: from evaluation to therapeutic management. Transpl Int (2024) 37:12803. 10.3389/ti.2024.12803 39416809 PMC11479921

[3] CDC. National Center for Health Statistics Mortality Data on CDC Wonder (2026). Available online at: https://wonder.cdc.gov/mcd.html.

[4] Eldin C Grossi PA Manda V Kamar N Lortholary O Hirsch HH Updates on donor-derived infection in solid organ transplantation, report from the 2024 GTI (infection and transplantation group) annual meeting. Transpl Int (2025) 38:14237. 10.3389/ti.2025.14237 40881322 PMC12380625

[5] Khan J Kozuch J Aslam S . Hepatitis B, C, and E viruses in solid organ transplantation: donor and recipient perspectives. JHLT Open (2026) 12:100533. 10.1016/j.jhlto.2026.100533 42005560 PMC13087687

[6] Lopez-Soler RI Joyce C Cotiguala L Aguirre O Samra M Trotter C Utilization of hepatitis B viremic donors (NAT+) leads to improved kidney transplant access for older adult recipients with little to no wait time. Transpl Infect Dis (2024) 26(3):e14295. 10.1111/tid.14295 38761060

[7] Croome KP Lee DD Pungpapong S Keaveny AP Taner CB . What are the outcomes of declining a public health service increased risk liver donor for patients on the liver transplant waiting list? Liver Transpl (2018) 24(4):497–504. 10.1002/lt.25009 29341398

[8] Mularoni A Cona A Bulati M Busà R Miele M Timoneri F Serologic screening and molecular surveillance of kaposi sarcoma herpesvirus/human herpesvirus-8 infections for early recognition and effective treatment of kaposi sarcoma herpesvirus-associated inflammatory cytokine syndrome in solid organ transplant recipients. Am J Transpl (2025) 25(5):1070–85. 10.1016/j.ajt.2024.11.013 39551265

[9] Ushiro-Lumb I Forsythe J Haywood B Geoghegan C Maddox V Ijaz S Impact of hepatitis E virus screening in the UK deceased organ donor population. Transpl Int (2023) 36:11673. 10.3389/ti.2023.11673 37727381 PMC10505649

[10] Peghin M Grossi PA . COVID-19 positive donor for solid organ transplantation. J Hepatol (2022) 77(4):1198–204. 10.1016/j.jhep.2022.06.021 35798131 PMC9251900

[11] Alcayna T Rao VB Lowe R . Identifying the climate sensitivity of infectious diseases: a conceptual framework. Lancet Planet Health (2025) 9(8):101291. 10.1016/j.lanplh.2025.101291 40763757

[12] Tan SSX Phoompoung P Okamoto K Chayakulkeeree M Koh XX Tan C Donor-derived infections-insights from Singapore, Japan, and Thailand. Transpl Infect Dis (2024) 26(Suppl. 1):e14370. 10.1111/tid.14370 39226139

[13] Levitsky J Formica RN Bloom RD Charlton M Curry M Friedewald J The American society of transplantation consensus conference on the use of hepatitis C viremic donors in solid organ transplantation. Am J Transpl (2017) 17(11):2790–802. 10.1111/ajt.14381 28556422

[14] Bari K Luckett K Kaiser T Diwan T Cuffy M Schoech MR Hepatitis C transmission from seropositive, nonviremic donors to non-hepatitis C liver transplant recipients. Hepatology (2018) 67(5):1673–82. 10.1002/hep.29704 29205441

[15] Zingg SW Lemon K . Donor viral hepatitis and liver transplantation. Surg Clin North Am (2024) 104(1):67–77. 10.1016/j.suc.2023.07.002 37953041

[16] Rivosecchi RM Sanchez PG Sacha LM Horn ET Keebler M Silveira FP . A single center experience of concomitant administration of sofosbuvir/velpatasvir and amiodarone after heart and lung transplantation. JHLT Open (2025) 9:100279. 10.1016/j.jhlto.2025.100279 40497232 PMC12150105

[17] IDSA. (2026). Available online at: https://www.hcvguidelines.org/ (Accessed June 30, 2026).

[18] Trapianti. Ora possibile l'utilizzo di organi di donatori con epatite C su riceventi negativi: via libera all'uso tempestivo delle nuove terapie. (2026). Available online at: https://www.trapianti.salute.gov.it/it/cnt-primopiano/ora-possibile-lutilizzo-di-organi-di-donatori-con-epatite-c-su-riceventi-negativi/ (Accessed September 22, 2025).

[19] Papanikolla J McGowan M Chunduru M Winters H Pesavento T Smith R Real-world experience in treatment of donor-derived hepatitis C virus in kidney transplant recipients with delayed initiation, shortened course glecaprevir/pibrentasvir *versus* standard of care. Transpl Infect Dis (2024) 26(6):e14366. 10.1111/tid.14366 39226149 PMC11666865

[20] Buchanan-Peart KA Pagan J Martin E Turkeltaub J Reese P Goldberg DS . Temporal changes in the utilization of kidneys from hepatitis C virus-infected donors in the United States. Am J Transpl (2023) 23(6):831–8. 10.1016/j.ajt.2023.03.001 36893936

[21] Stewart ZA Stern J Ali NM Kalia HS Khalil K Jonchhe S Clinical and financial implications of 2 treatment strategies for donor-derived hepatitis C infections. Transpl Direct (2021) 7(10):e762. 10.1097/TXD.0000000000001222 34514117 PMC8425828

[22] Mazur M Pisani B Carmona Rubio A Eisen HJ Bhat G Nunez M . Early outcomes in patients undergoing heart re-transplantation in the era of HCV-viremic donors. J Cardiol (2025) 87:340–6. 10.1016/j.jjcc.2025.09.005 40967272

[23] Addison V Goldberg DS Vianna R Martin E Garcia J . Multivisceral transplantation utilizing hepatitis C virus-viremic donors for hepatitis C virus-negative recipients. Am J Transpl (2025) 25(1):204–8. 10.1016/j.ajt.2024.09.006 39277142

[24] Khan S Mazumder R Wang X Wang Y Sims OT Budev M Hepatitis C viremic lung transplantation to aviremic recipients: comprehensive outcomes and post-transplant viremia. Clin Transpl (2024) 38(5):e15325. 10.1111/ctr.15325 38716770

[25] Kwon JH Hill MA Patel R Tedford RJ Hashmi ZA Shorbaji K Outcomes of over 1000 heart transplants using hepatitis C-Positive donors in the modern era. Ann Thorac Surg (2023) 115(2):493–500. 10.1016/j.athoracsur.2022.11.002 36368348

[26] Schlendorf KH Zalawadiya S Shah AS Perri R Wigger M Brinkley DM Expanding heart transplant in the era of direct-acting antiviral therapy for hepatitis C. JAMA Cardiol (2020) 5(2):167–74. 10.1001/jamacardio.2019.4748 31851352 PMC6990812

[27] Pratt CG Noriega N Whitrock JN Carter MM Moore AN Kaiser TE Follow up and safety of use of hepatitis C virus discordant liver transplants. HPB (Oxford) (2025) 28:524–33. 10.1016/j.hpb.2025.12.027 41486031

[28] Nakayama T Krist DT Akabane M Imaoka Y Esquivel CO Kwong AJ Hepatitis C-positive grafts for hepatitis C-negative recipients in liver transplantation: buried treasure or depleting resource? Liver Transpl (2025) 31(9):1154–64. 10.1097/LVT.0000000000000557 39679922

[29] Paternostro K Birs A Aslam S Adler E Hong K Wettersten N . Intermediate-term risk of cardiac allograft vasculopathy following heart transplantation from hepatitis C viremic donors in the era of direct-acting antiviral therapy. JHLT Open (2026) 11:100416. 10.1016/j.jhlto.2025.100416 41312400 PMC12651725

[30] Woolley AE Gandhi AR Jones ML Kim JJ Mallidi HR Givertz MM The cost-effectiveness of transplanting hearts from Hepatitis C-infected donors into uninfected recipients. Transplantation (2023) 107(4):961–9. 10.1097/TP.0000000000004378 36525554 PMC10065819

[31] Russo FP Vigano M Stock P Ferrarese A Pugliese N Burra P HBV-positive and HIV-positive organs in transplantation: a clinical guide for the hepatologist. J Hepatol (2022) 77(2):503–15. 10.1016/j.jhep.2022.03.007 35398460

[32] Len O Los-Arcos I Aguado JM Blanes M Bodro M Carratalà J Selection criteria of solid organ donors in relation to infectious diseases: a Spanish consensus. Transpl Rev (Orlando) (2020) 34(2):100528. 10.1016/j.trre.2020.100528 32001103

[33] Peel J Tang MJ Morrissey CO . Hepatitis B in transplant recipients: an Australian single-centre study of screening, vaccination and management. Intern Med J (2025) 55(11):1824–31. 10.1111/imj.70202 41014282

[34] Elharabi Z Saba J Akin H . Navigating the latest Hepatitis B virus reactivation guidelines. Diseases (2025) 13(11):355. 10.3390/diseases13110355 41294895 PMC12651630

[35] Alvarez-Lopez P Riveiro-Barciela M Oleas-Vega D Flores-Cortes C Román A Perelló M Anti-HBc impacts on the risk of hepatitis B reactivation but not on survival of solid-organ transplant recipients. Medicine (Baltimore) (2020) 99(9):e19407. 10.1097/MD.0000000000019407 32118794 PMC7478413

[36] Yin S Chen X Li X Zhang F Wu J Lin T . Was antiviral prophylaxis necessary after kidney transplantation utilizing HBcAb+ donors? A systematic review and meta-analysis. Transpl Rev (Orlando) (2024) 38(2):100840. 10.1016/j.trre.2024.100840 38489866

[37] Belga S Wright RC Lee SB Durand CM Doucette K . Hepatitis B virus donor positive to recipient negative (D+/R-) heart and lung transplantation: analysis from the organ procurement and transplantation network. Transpl Infect Dis (2025) 27(5):e70054. 10.1111/tid.70054 40693753 PMC12519922

[38] Vinaixa C DiMaira T Russo FP Goldberg D Mazzola A Walabh P Use of HBsAg-positive donors in liver transplantation: an ILTS-EASL-AASLD multisociety survey. Liver Transpl (2024) 30(11):1116–22. 10.1097/LVT.0000000000000432 39018028

[39] Tam PCK Katz-Greenberg G Wolfe CR Lott K Berg CL DeVore AD Hepatitis B virus NAT positive donors in non-hepatic organ transplant: quantifying viral loads to optimize recipient risk stratification and management in the prevention of donor-derived infection. Clin Transpl (2025) 39(7):e70236. 10.1111/ctr.70236 40698537 PMC12284844

[40] European Association for the Study of the L Sandmann L Jaroszewicz J Kennedy P Lampertico P Lemoine M EASL clinical practice guidelines on the management of hepatitis B virus infection. J Hepatol (2025) 83(2):502–83. 10.1016/j.jhep.2025.03.018 40348683

[41] Ghany MG Pan CQ Lok AS Feld JJ Lim JK Wang SH AASLD ISDA practice guideline on treatment of chronic hepatitis B. Hepatology (2026) 83(4):974–97. 10.1097/HEP.0000000000001549 41186418

[42] Franchello A Ghisetti V Marzano A Romagnoli R Salizzoni M . Transplantation of hepatitis B surface antigen-positive livers into hepatitis B virus-positive recipients and the role of hepatitis Delta coinfection. Liver Transpl (2005) 11(8):922–8. 10.1002/lt.20471 16035057

[43] Angelico R Trapani S Manzia TM Lenci I Grossi P Ricci A Liver transplantation for hepatitis D virus/hepatitis B virus coinfection in Italy: an intention-to-treat analysis of long-term outcomes. Am J Transpl (2025) 25(7):1502–14. 10.1016/j.ajt.2025.03.003 40057194

[44] Martini S Tandoi F Romagnoli R Rizzetto M . Liver transplantation in hepatitis B/Hepatitis D (delta) virus coinfected recipients. Transplantation (2022) 106(10):1935–9. 10.1097/TP.0000000000004138 35404869

[45] Wedemeyer H Aleman S Brunetto MR Blank A Andreone P Bogomolov P A phase 3, randomized trial of bulevirtide in chronic hepatitis D. N Engl J Med (2023) 389(1):22–32. 10.1056/NEJMoa2213429 37345876

[46] Chiu CY Sampathkumar P Brumble LM Vikram HR Watt KD Beam E . Hepatitis B vaccine compliance, serologic response, and durability in adult thoracic organ transplant recipients. Clin Transpl (2024) 38(9):e15464. 10.1111/ctr.15464 39302222

[47] Hansrivijit P Trongtorsak A Puthenpura MM Boonpheng B Thongprayoon C Wijarnpreecha K Hepatitis E in solid organ transplant recipients: a systematic review and meta-analysis. World J Gastroenterol (2021) 27(12):1240–54. 10.3748/wjg.v27.i12.1240 33828397 PMC8006097

[48] Sridhar S Cheng VCC Wong SC Yip CCY Wu S Lo AWI Donor-derived genotype 4 hepatitis E virus infection, Hong Kong, China, 2018. Emerg Infect Dis (2019) 25(3):425–33. 10.3201/eid2503.181563 30789146 PMC6390757

[49] Binda B Picchi G Bruni R Di Gasbarro A Madonna E Villano U The prevalence, risk factors, and outcomes of hepatitis E virus infection in solid organ transplant recipients in a highly endemic area of Italy. Viruses (2025) 17(4):502. 10.3390/v17040502 40284945 PMC12031106

[50] Lhomme S Bardiaux L Abravanel F Gallian P Kamar N Izopet J . Hepatitis E virus infection in solid organ transplant recipients, France. Emerg Infect Dis (2017) 23(2):353–6. 10.3201/eid2302.161094 28098552 PMC5324811

[51] Abravanel F Lhomme S Marion O Peron JM Kamar N Izopet J . Diagnostic and management strategies for chronic hepatitis E infection. Expert Rev Anti Infect Ther (2023) 21(2):143–8. 10.1080/14787210.2023.2166932 36625025

[52] Riveiro-Barciela M Roade L Martinez-Camprecios J Vidal-González J Rodríguez-Diez B Perelló M mTOR inhibitors a potential predisposing factor for chronic hepatitis E: results from the prospective collaborative CHES study (chronic hepatitis EScreening in patients with immune impairment and increased transaminases levels). Gastroenterol Hepatol (2023) 46(10):764–73. 10.1016/j.gastrohep.2023.01.010 36731726

[53] Huang S Zhang X Su Y Zhuang C Tang Z Huang X Long-term efficacy of a recombinant hepatitis E vaccine in adults: 10-Year results from a randomised, double-blind, placebo-controlled, phase 3 trial. Lancet (2024) 403(10429):813–23. 10.1016/S0140-6736(23)02234-1 38387470

[54] Gray ML Tingle SJ Elzawahry M Hurn E Mahendran B Post FA The impact of recipient HIV status on kidney transplant outcomes in the United Kingdom: have previous studies overestimated risk? Transplantation (2026) 110(3):e670–e679. 10.1097/TP.0000000000005585 41332243 PMC12908647

[55] Miro JM Amondarain NL Serrano L Salmanton-Garcia J Manzardo C Malano-Barletta D Current situation of solid organ transplantation from HIV-positive donors to HIV-positive recipients in Europe: the Spanish perspective. Clin Microbiol Infect (2026) 32(1):137–46. 10.1016/j.cmi.2025.08.025 40907699

[56] Griffin D Vaz K Basu G Mulley W Kotecha S Levin K Knowledge and attitudes about solid organ transplantation for people with HIV in Australia: a cross-sectional survey of people with and without HIV. J Acquir Immune Defic Syndr (2026) 101(4):432–40. 10.1097/QAI.0000000000003815 41416806

[57] Rozek GM Yang P Eby Y Benner SE Martens C Habtehyimer F HIV superinfection in kidney transplant recipients with HIV who received organs from donors with HIV. J Infect Dis (2025) 232(2):359–63. 10.1093/infdis/jiaf284 40439124 PMC12349943

[58] Saeed O Hemmige V Angelo L Gjelaj C Puius YA Madan S Early experience in heart transplantation utilizing donors with HIV. J Heart Lung Transpl (2026) 45(3):468–72. 10.1016/j.healun.2025.09.011 40998274

[59] Balachandran IC Adekunle RO . A new frontier of HIV organ policy equity - expanding the transplant donor pool. J Heart Lung Transpl (2026) 45(3):473–4. 10.1016/j.healun.2025.10.030 41238003

[60] Grossi PA Grossi AA . Solid organ transplantation from HIV-Positive donors to HIV-positive recipients in Europe: a growing opportunity. Clin Microbiol Infect (2026) 32(1):16–8. 10.1016/j.cmi.2025.11.001 41213326

[61] Botha J Conradie F Etheredge H Fabian J Duncan M Haeri Mazanderani A Living donor liver transplant from an HIV-positive mother to her HIV-negative child: opening up new therapeutic options. AIDS (2018) 32(16):F13–F19. 10.1097/QAD.0000000000002000 30281558 PMC6200383

[62] Mularoni A Cona A Mikulska M Pecoraro F Piazza C De Vita E HHV-8/KSHV in solid organ transplantation: current gaps of knowledge and future directions. Transpl Infect Dis (2026) 28:e70179. 10.1111/tid.70179 41615270 PMC13070012

[63] Nambiar P Liang T Labo N Hand J Blumberg EA Rana MM Kaposi sarcoma-associated herpesvirus risk and disease in kidney donors and transplant recipients with HIV in the United States. Clin Infect Dis (2025) 82:709–19. 10.1093/cid/ciaf229 40324947 PMC13110356

[64] Lombardi A . Molecular respiratory pathogen panels in lung transplantation. Transpl Infect Dis (2026) 28(1):e70119. 10.1111/tid.70119 41450121 PMC12892826

[65] Lombardi A Renisi G Liparoti A Bobbio C Bandera A . Application of syndromic panels for respiratory tract infections in lung transplantation: a critical review on current evidence and future perspectives. Transpl Infect Dis (2026) 28(1):e14448. 10.1111/tid.14448 39888105 PMC12892830

[66] Gardiner BJ Ison MG . Donor-derived infections in heart and lung transplant recipients. JHLT Open (2025) 10:100376. 10.1016/j.jhlto.2025.100376 40979522 PMC12444161

[67] Asija R Singh R Paneitz DC Wolfe SB Chukwudi C Michel E Is transplantation with coronavirus disease 2019-Positive donor lungs safe? A US nationwide analysis. Ann Thorac Surg (2023) 116(5):1046–54. 10.1016/j.athoracsur.2023.05.048 37506993

[68] Peghin M Graziano E De MM Balsamo ML Isola M López-Fraga M Acceptance of organs from deceased donors with resolved or active SARS-CoV-2 infection: a survey from the council of Europe. Transpl Int (2024) 37:13705. 10.3389/ti.2024.13705 39640248 PMC11617184

[69] Cockbain AJ Jacob M Ecuyer C Hostert L Ahmad N . Transplantation of solid organs procured from influenza A H1N1 infected donors. Transpl Int (2011) 24(12):e107–10. 10.1111/j.1432-2277.2011.01342.x 21954984

[70] Petrisli E Gabrielli L De Cillia C Liberatore A Piccirilli G Venturoli S Molecular testing in organ biopsies and perfusion fluid samples from severe acute respiratory syndrome coronavirus 2 positive donors. Viruses (2025) 17(12):1611. 10.3390/v17121611 41472280 PMC12737485

[71] Kothadia SM Wolfe CR Baker AW Young KA Reynolds JM Hartwig MG Outcomes of lung transplantation from SARS-CoV-2 positive donors during the omicron wave. JHLT Open (2025) 8:100249. 10.1016/j.jhlto.2025.100249 40242055 PMC12002999

[72] Goren LR Adeyi O Thielen BK . Possible donor-derived infection in a pediatric liver transplant patient with granulomatous hepatitis. Cureus (2023) 15(11):e49136. 10.7759/cureus.49136 38130518 PMC10733164

[73] Pettengill MA Babu TM Prasad P Chuang S Drage MG Menegus M Probable donor-derived human adenovirus type 34 infection in 2 kidney transplant recipients from the same donor. Open Forum Infect Dis (2019) 6(3):ofy354. 10.1093/ofid/ofy354 30882008 PMC6411205

[74] Kaul DR Vece G Blumberg E La Hoz RM Ison MG Green M Ten years of donor-derived disease: a report of the disease transmission advisory committee. Am J Transpl (2021) 21(2):689–702. 10.1111/ajt.16178 32627325

[75] Multidisciplinary expertise at the service of public health. (2026). Available online at: https://www.hcsp.fr (Accessed July 18, 2024).

[76] Peghin M Hirsch HH Len O Codina G Berastegui C Sáez B Epidemiology and immediate indirect effects of respiratory viruses in lung transplant recipients: a 5-Year prospective study. Am J Transpl (2017) 17(5):1304–12. 10.1111/ajt.14042 27615811 PMC7159570

[77] Narsana N Ha D Ho DY . Treating adenovirus infection in transplant populations: therapeutic options beyond cidofovir? Viruses (2025) 17(5):599. 10.3390/v17050599 40431613 PMC12116135

[78] Meylan PR Aubert JD Kaiser L . Influenza transmission to recipient through lung transplantation. Transpl Infect Dis (2007) 9(1):55–7. 10.1111/j.1399-3062.2006.00175.x 17313474

[79] Le Page AK Kainer G Glanville AR Tu E Bhonagiri D Rawlinson WD . Influenza B virus transmission in recipients of kidney and lung transplants from an infected donor. Transplantation (2010) 90(1):99–102. 10.1097/TP.0b013e3181da1933 20606569

[80] Organ donation and seasonal influenza: guidance from SaBTO's virology review subcommittee. Available online at: https://www.gov.uk/government/publications/organ-donation-and-seasonal-flu/organ-donation-and-seasonal-influenza-guidance-from-sabtos-virology-review-subcommittee (Accessed October 23, 2019).

[81] Hulot S Prunet V Coiffard B Zandotti C Boschi C D'Journo XB Fatal acute respiratory distress syndrome caused by donor-derived human metapneumovirus after lung transplantation. Am J Transpl (2025) 25(10):2239–43. 10.1016/j.ajt.2025.05.020 40389161

[82] Nellore A Goepfert P Tan CS Bajema K Belden K Blumberg D IDSA 2025 guidelines on the use of vaccines for the prevention of seasonal COVID-19, influenza, and RSV infections in immunocompromised patients. Clin Infect Dis (2026) 82:i105–i110. 10.1093/cid/ciag114 41766454

